# Ovarian borderline mucinous tumor with carcinosarcomatous mural nodule: a case report of a rare entity and review of the literature

**DOI:** 10.3389/fmed.2026.1826883

**Published:** 2026-04-28

**Authors:** Yuming Bai, Yan Xia

**Affiliations:** Cancer Center, Department of Pathology, Zhejiang Provincial People’s Hospital, Affiliated People’s Hospital, Hangzhou Medical College, Hangzhou, Zhejiang, China

**Keywords:** carcinosarcoma, mural nodule, ovarian borderline mucinous tumor, pathology, rare tumors

## Abstract

**Background:**

Ovarian mucinous tumors are histologically classified into benign, borderline, and malignant categories, with borderline tumors generally demonstrating an intermediate prognosis. However, ovarian borderline mucinous tumors with malignant mural nodules are associated with poor prognosis, where sarcomatous and anaplastic carcinoma mural nodules are common, whereas carcinosarcomatous mural nodules are rare.

**Case presentation:**

This report describes a case of a 27-year-old female patient presenting with a two-year history of abdominal distension. Imaging studies revealed a large cystic-solid mass with abnormal signal characteristics. The patient underwent exploratory laparotomy combined with fertility-sparing staging surgery for suspected ovarian malignancy. Postoperative pathological diagnosis confirmed ovarian borderline mucinous tumor with carcinosarcomatous mural nodule. The patient subsequently received adjuvant chemotherapy, and at 5.5 months of follow-up, there has been no evidence of recurrence.

**Conclusion:**

This case provides novel insights into the diagnosis of ovarian borderline mucinous tumor with a carcinosarcomatous mural nodule, contributing to an enhanced understanding and recognition of carcinosarcomatous mural nodules.

## 1 Introduction

Mucinous ovarian tumors (MOTs) are a subtype of ovarian epithelial neoplasms, accounting for approximately 10 to 15% of all ovarian tumors. They predominantly affect women aged 20 to 40 years (1). Histopathologically, MOTs are classified into benign, borderline, and malignant categories (2). Ovarian borderline tumors are defined as neoplasms of low malignant potential with biological behavior intermediate between benign and malignant conditions, accounting for approximately 15% of all ovarian tumors (3). Based on the type of surface epithelium, these tumors are classified into borderline serous tumors and borderline mucinous tumors. Mural nodules are one or more solid areas that arise within ovarian tumors (4) and are histologically distinct from the primary tumor. These nodules frequently arise in ovarian mucinous tumors, with borderline mucinous tumors and mucinous carcinomas being the predominant tumor types. This article reports a rare case of ovarian borderline mucinous tumor with a malignant mural nodule, in which the mural nodule was pathologically diagnosed as carcinosarcoma.

## 2 Case presentation

**Figure 1:**
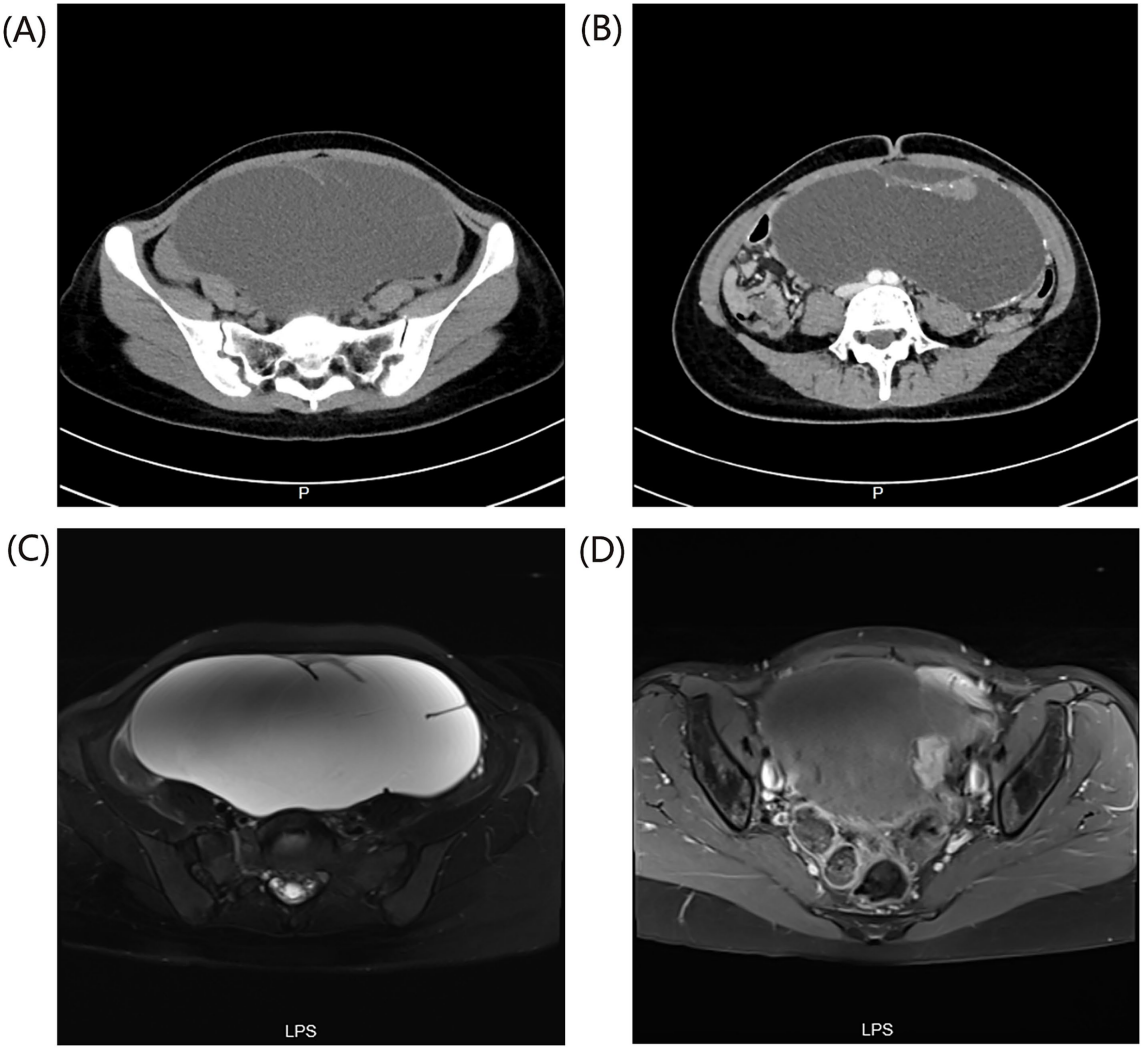
Pelvic imaging findings. **(A)** A large cystic lesion is observed in the abdominopelvic cavity. **(B)** Punctate calcifications are noted along the lesion margins and septa, with enhancement evident in the septa and solid components. **(C)** A large cystic-solid lesion with abnormal signal intensity is observed in the pelvis. **(D)** On T2WI, the cystic component demonstrates high signal intensity, the solid component shows iso- to hypointense signal, and focal diffusion restriction is evident in the solid portion.

The patient is a 27-year-old female, residing in a city in Zhejiang Province, China. She presented with a 2-year history of increased abdominal girth. No prior medical intervention or treatment was sought during this period. There was no known family history of similar conditions, genetic disorders, or infectious diseases among first-degree relatives. The patient had no significant past medical history. No history of hypertension, diabetes, or chronic illnesses. No prior surgeries, hospitalizations, or blood transfusions. No known drug allergies. Pelvic computed tomography (CT) scan revealed a large cystic lesion in the abdominopelvic cavity (Figure 1A), measuring approximately 275 mm × 193 mm × 120 mm, with septations and small solid soft tissue components. Punctate calcifications were observed at the lesion margins and septations. On contrast-enhanced scanning, the lesion showed no significant enhancement, while the septations and solid portions demonstrated enhancement (Figure 1B); adjacent bowel loops were displaced. The findings were suggestive of an ovarian cystadenoma. Pelvic magnetic resonance imaging (MRI) showed a large cystic-solid abnormal signal shadow in the pelvis (Figure 1C), with irregular shape and clear boundaries, and a cross-sectional size of approximately 191 mm × 72 mm. The mural nodule measured approximately 27 mm × 23 mm. On T1-weighted imaging (T1WI), it exhibited low signal intensity with small foci of high signal intensity; on T2-weighted imaging (T2WI), the cystic portion displayed high signal intensity, whereas the solid portion showed iso- to low signal intensity. Localized restricted diffusion was noted in the solid portion. On contrast-enhanced scanning, significant enhancement was observed in the solid portion (Figure 1D), while the cystic portion showed no enhancement. Adjacent tissues were compressed and displaced, indicating a neoplastic lesion. Serum tumor marker tests showed: carbohydrate antigen CA15-3: 9.7 U/mL; neuron-specific enolase (NSE): 11.8 ng/mL; squamous cell carcinoma antigen: 0.6 ng/mL; cytokeratin 19 fragment (CYFRA 21-1): 2.7 ng/mL; carbohydrate antigen CA72-4: 16.6 U/mL; human epididymis protein 4 (HE4): 47.2 pmol/L; pro-gastrin-releasing peptide (ProGRP): 45.0 pg/mL; alpha-fetoprotein (AFP): 6.5 μg/L; carcinoembryonic antigen (CEA): <1.7 μg/L; carbohydrate antigen CA125: 29.1 U/mL; carbohydrate antigen CA19-9: <2.1 U/mL; all within normal limits.

On the third day after admission, the patient underwent fertility-sparing comprehensive surgical staging via exploratory laparotomy. The procedure included left salpingo-oophorectomy, partial omentectomy, peritoneal lesion excision, excision of pelvic endometriosis, lysis of adhesions, pelvic lymphadenectomy, fulguration of endometriotic lesions, and pelvic peritonectomy. Intraoperative exploration revealed an irregular, cystic mass measuring approximately 27 × 19 × 12 cm in the left adnexa. The mass was gourd-shaped with a smooth surface and soft consistency, showing no obvious adhesions to surrounding tissues. The right adnexa and uterus appeared normal. Adhesions were noted between the right abdominal wall and the intestines. Multiple black endometriotic lesions were identified on the bilateral uterosacral ligaments, the anterior and posterior leaves of the broad ligaments, the pouch of Douglas, and the uterine surface. Several mildly enlarged lymph nodes were palpated in the pelvis. No abnormalities were observed on the surface of the remaining intestines, appendix, liver, or other vital organs. Upon puncture, the mass released a large volume of yellow fluid, followed by a small amount of brownish fluid.

**Figure 2:**
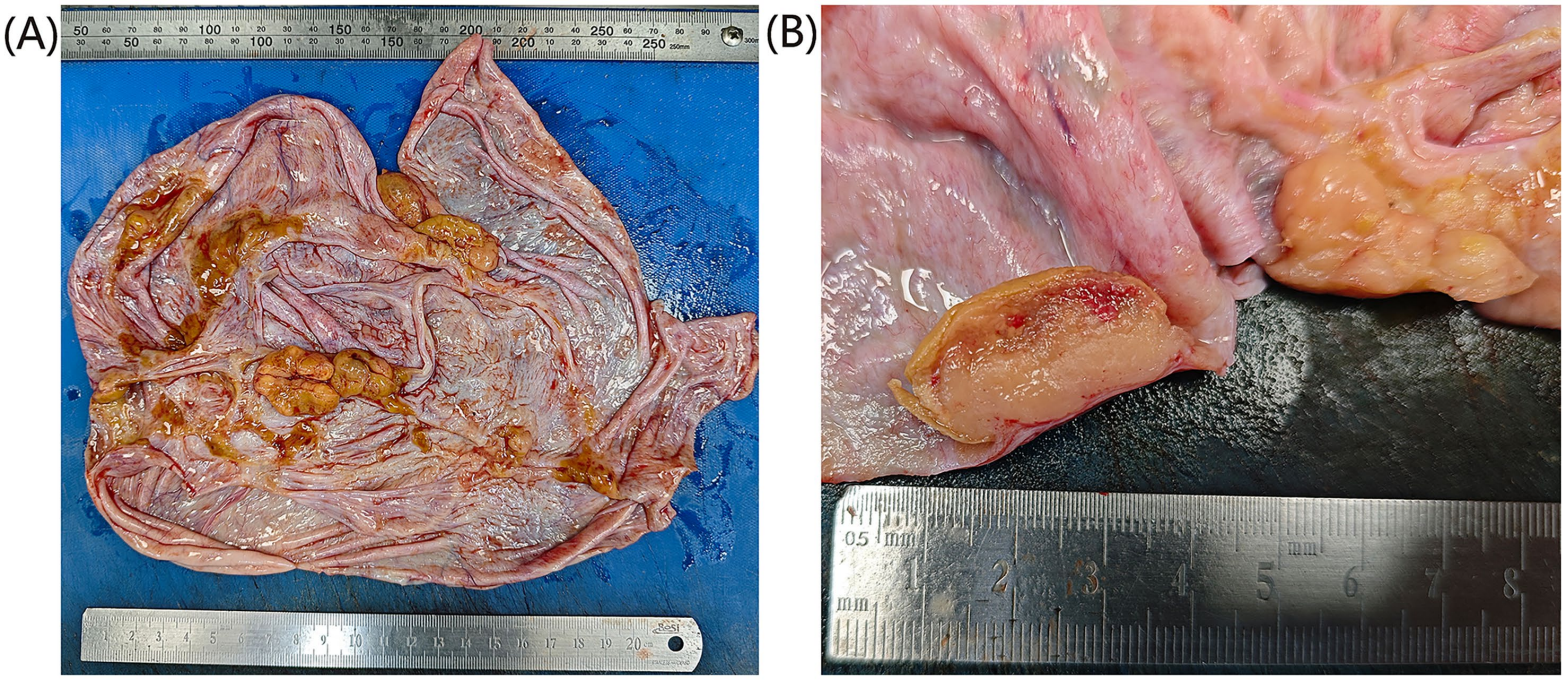
Gross pathological specimen. **(A)** A large cyst with multiple mural nodules (1–3.5 cm in diameter) was observed. **(B)** The mural nodules exhibited a yellowish-gray cut surface and firm consistency, and were well-demarcated from the surrounding tissue.

**Figure 3:**
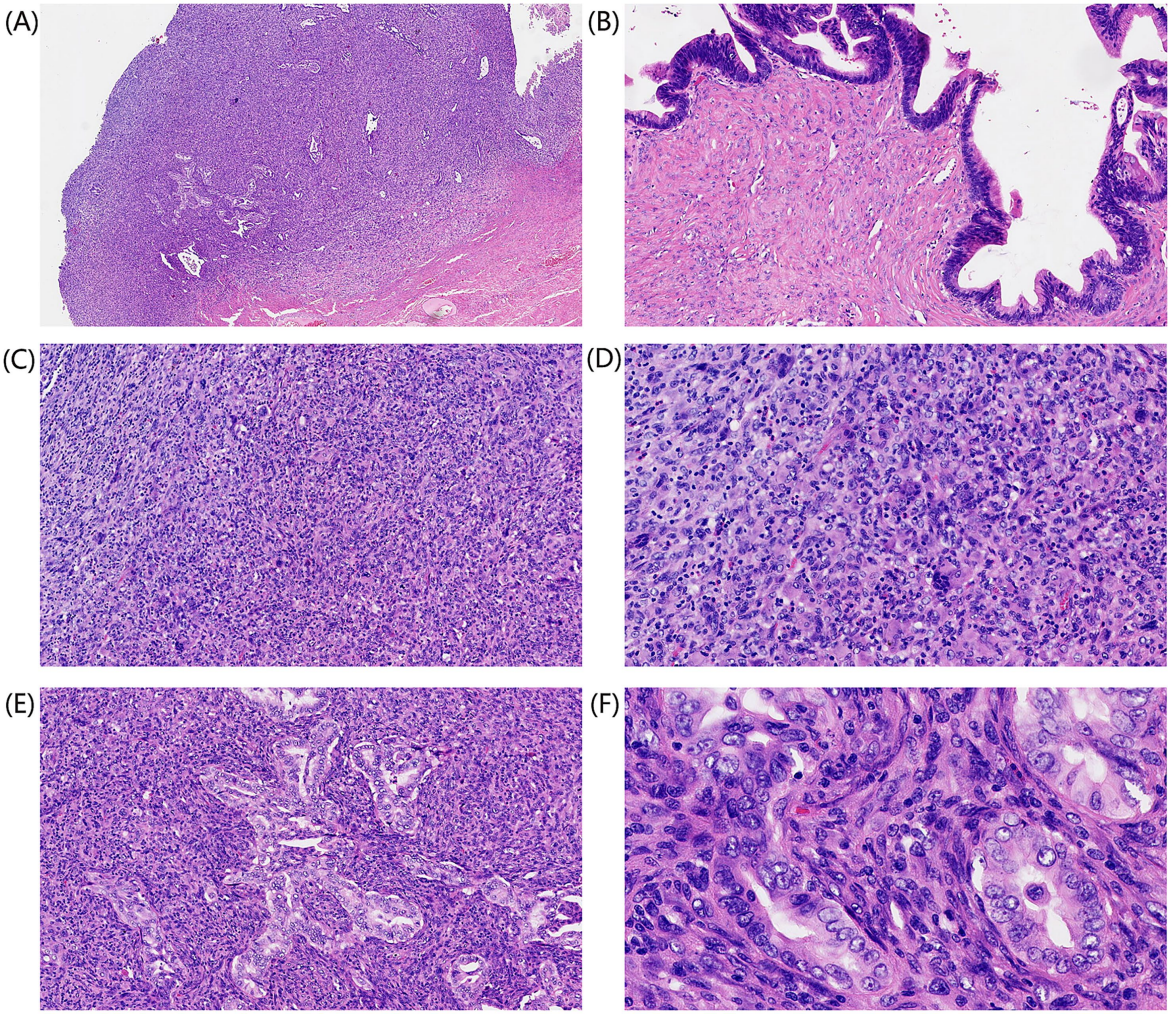
Histopathological features. **(A)** Mural nodule (20×). **(B)** The cyst wall is lined by gastrointestinal-type mucinous epithelium (100×). **(C)** Numerous short spindle-shaped cells are densely arranged in solid sheet-like structures (100×). **(D)** The background shows multinucleated giant cells, eosinophil infiltration, and plasma cell infiltration (200×). **(E)** Glandular structures are interspersed within the spindle cell areas (100×). **(F)** Cells exhibit eosinophilic staining, clear cytoplasm, and moderate nuclear atypia (400×).

Gross pathological findings: A piece of whitish-yellowish cystic tissue measuring 16 × 11 × 2 cm, clinically incised with loss of cystic contents (Figure 2A). Multiple yellowish nodules (1–3.5 cm in diameter) are attached to the cyst wall, exhibiting a solid, whitish-yellowish cut surface of moderate firmness, and were well-demarcated from the surrounding tissue (Figure 2B). Additionally, a fallopian tube segment (13 cm in length, 0.7–1.0 cm in diameter) with an intact fimbriated end is provided. Histopathological examination: Microscopically, the cyst wall is lined by intestinal-type mucinous epithelium with mild to moderate atypia. Cells exhibit crowded arrangement, pseudostratification, and focal tubular structures. Focal cell clusters are observed without definite stromal invasion. The mural nodules consist of two distinct components (Figures 3A,B). Component 1: Dense solid sheets of short spindle-shaped cells with marked cellular atypia and frequent mitotic figures. Multinucleated giant cells, eosinophils, plasma cells, and lymphocytic infiltration are present in the background. No vascular invasion is noted (Figures 3C,D). Component 2: Central regions of the mural nodules show irregular glandular tubular structures intermingled with spindle cell areas. Glands display eosinophilic cells with clear cytoplasm and moderately atypical nuclei (Figures 3E,F).

**Figure 4:**
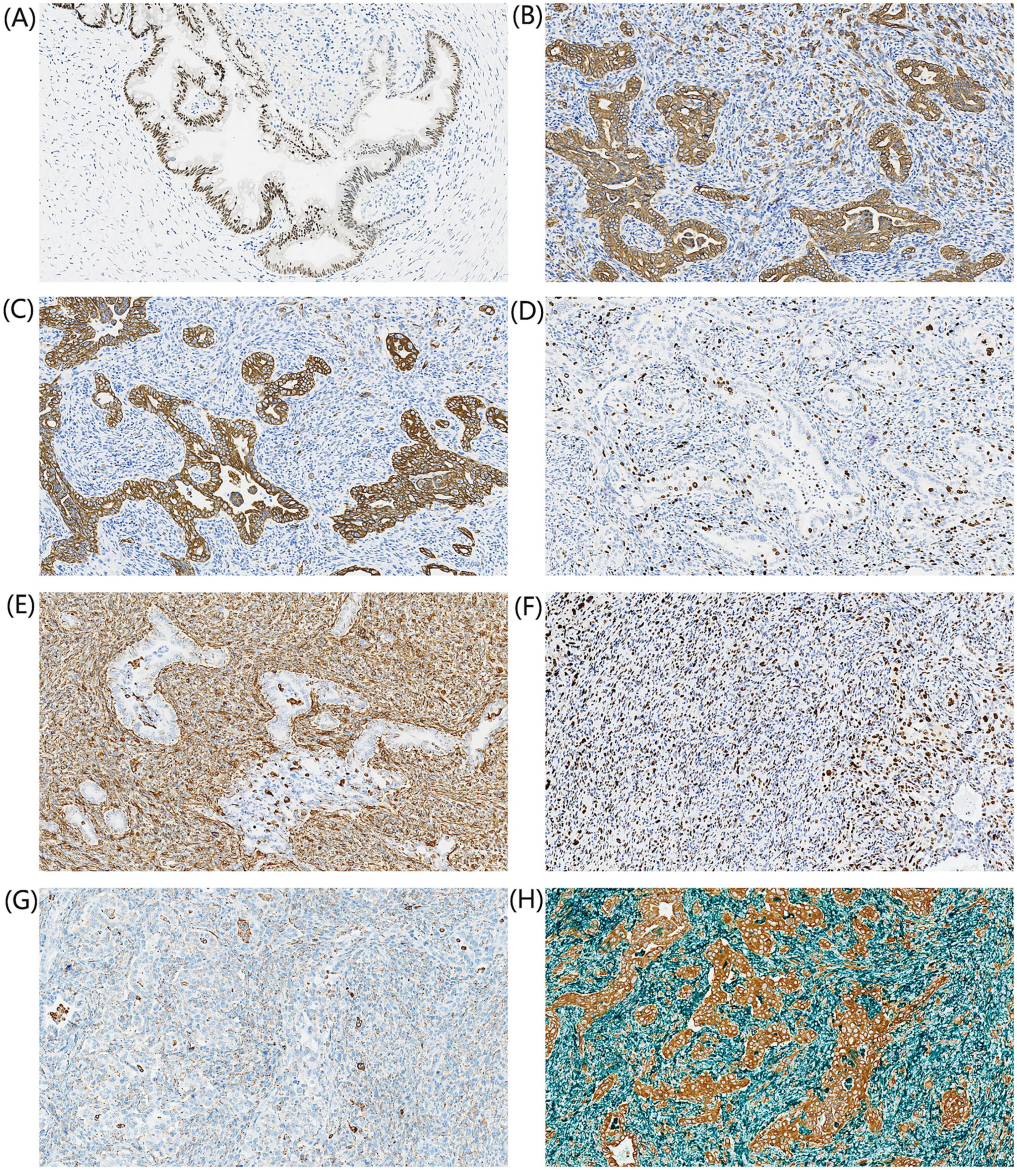
Immunohistochemical findings. **(A)** Mucinous epithelium in the cyst wall region demonstrates CDX2 expression (100×). **(B,C)** Epithelial regions show positivity for CK(Pan) and CK7(100×). **(D)** The Ki-67 labeling index was approximately 20% in the glandular epithelial component and 30% in the spindle cell component (100×). **(E)** Spindle cells exhibit vimentin expression (100×). **(F)** Strong p53 expression is evident in spindle cells (100×). **(G)** The background area reveals scattered CD68-positive cells (100×). **(H)** CK(Pan)/vimentin immunohistochemical double staining is displayed (100×).

Immunohistochemical staining revealed that in the cyst wall area (Figure 4A), the mucinous epithelium exhibited positivity for cytokeratin 7 (CK7), caudal type homeobox 2 (CDX2), and focal positivity for cytokeratin 20 (CK20); in the glandular epithelial area of the mural nodule (Figures 4B,D), cells were positive for pan-cytokeratin (CK(Pan)), CK7, epithelial membrane antigen (EMA), villin, and Ki-67 antigen (Ki67) (approximately 20%); in the spindle cell area (Figures 4E,F), cells showed positivity for vimentin, strong positivity for tumor protein p53 (p53), partial positivity for smooth muscle actin (SMA), focal positivity for special AT-rich sequence-binding protein 2 (SATB2) and S100 protein, with Ki67 positivity in approximately 30% of cells, and negativity for desmin, myogenin, cluster of differentiation 34 (CD34), calponin, human melanoma black 45 (HMB45), paired box 8 (PAX-8), wilms tumor 1 (WT1), estrogen receptor (ER), and progesterone receptor (PR); and in the stromal background area, a few cells were positive for cluster of differentiation 68 (CD68) (Figure 4G). Additionally, immunohistochemical double staining for CK(Pan)/vimentin demonstrated CK(Pan) positivity in the glandular area and vimentin positivity in the spindle cell area, with focal CK(Pan) positivity also observed within the spindle cell component (Figure 4H).

Based on histomorphological and immunohistochemical characteristics, the final diagnosis is ovarian borderline mucinous tumor with malignant mural nodule, where the mural nodule component is identified as carcinosarcoma. FIGO staging is stage IA. No cancer metastasis was observed in the patient’s regional lymph nodes (0/7), including the left internal iliac-obturator lymph node, left external iliac lymph node, and left common iliac lymph node. Intraoperative biopsies of the uterine surface, left sacrouterine ligament peritoneum, right sacrouterine ligament peritoneum, rectouterine pouch peritoneum, left broad ligament, bladder reflection peritoneum, left paracolic gutter peritoneum, right paracolic gutter peritoneum, greater omentum, and rectouterine pouch peritoneum all showed no tumor involvement. Postoperative adjuvant chemotherapy was initiated on day 14 following surgery. The patient completed 6 cycles of the TC regimen, comprising liposomal paclitaxel (240 mg) and carboplatin (550–700 mg), administered via intravenous infusion every 22 days. Follow-up assessments were conducted at 2.5 and 5.5 months postoperatively. Enhanced CT at 2.5 months revealed post-surgical changes with minimal pelvic effusion but no evidence of recurrent masses. Pelvic ultrasound at 5.5 months showed only a small anechoic area (depth: 2 cm) without solid lesions. Serum tumor markers remained stable or decreased throughout the follow-up period. Specifically, CA125 levels decreased from 11.3 U/mL to 6.5 U/mL, and HE4 levels decreased from 104.0 pmol/L to 75.1 pmol/L. All other markers (including CEA, CA19-9, AFP, etc.) remained within normal limits.

## 3 Discussion

Ovarian borderline mucinous tumor is a type of neoplasm with low malignant potential and favorable prognosis (5). However, when accompanied by mural nodules, the prognosis depends on the histological features of the nodules, and malignant mural nodules often require additional neoadjuvant therapy. Therefore, determining the histological type of mural nodules is crucial. Mural nodules are primarily classified into two major categories: benign and malignant (6).

Benign mural nodules typically exhibit a histologic pattern of reactive sarcomatoid proliferation, and are thus also referred to as sarcoma-like mural nodules (SLMN). SLMN generally exhibit a favorable prognosis and display reactive lesion characteristics, such as small size, well-demarcated borders, absence of vascular invasion or surrounding tissue invasion. SLMN are composed of a very cellular and heterogenous population of fibroblasts and inflammatory cells. Mitosis may be conspicuous in the fibrohistiocytic cells (7). Additionally, the background frequently shows histiocytes, lymphocytes, neutrophils, and scattered multinucleated giant cells, with hemorrhage commonly observed (8).

Malignant mural nodules are associated with poor prognosis. The mortality rate within 18 months reaches as high as 43% (9), whereas the 5-year survival rate for patients with FIGO stage I mucinous ovarian carcinoma is 91%. Consequently, meticulous identification and classification of mural nodules are essential for initiating earlier neoadjuvant therapy and improve patient outcomes. Malignant mural nodules associated with ovarian mucinous borderline tumors are rare entities with distinct pathological features. According to the WHO Classification of Female Genital Tumours (5th edition), these nodules are classified into anaplastic carcinoma, carcinosarcoma, and sarcoma subtypes (10). Most patients with anaplastic carcinoma mural nodules exhibit rapid disease progression and a poor prognosis. Barron-Cervantes et al. (11) reported a case of a female patient with ovarian borderline mucinous tumor accompanied by an anaplastic carcinoma mural nodule who died of renal failure two months postoperatively. Anaplastic carcinoma nodules are typically poorly demarcated, large, invasive lesions that may or may not involve lymphatic invasion, usually composed of polygonal cells with high-grade nuclear features; however, inflammatory infiltration is often minimal (12). Immunohistochemically, these nodules characteristically demonstrate diffuse strong positive expression of CK and negative expression of vimentin (13). Sarcomatous nodules are characterized by large size (tumor diameter frequently exceeding 10 cm), prominent hemorrhage and necrosis, poorly demarcated borders, and vascular invasion—which serves as a reliable diagnostic criterion for true malignant tumors. Tumor cells exhibit diverse morphology, commonly containing mononuclear or multinucleated malignant cells, giant cells, abnormal pathological mitotic figures, and marked pyknosis, with minimal inflammatory cell infiltration (14).

Ovarian borderline mucinous tumor with a carcinosarcomatous mural nodule represents a rare entity characterized by aggressive potential. The diagnostic cornerstone entails confirming the coexistence of a mucinous borderline tumor background and a biphasic malignant mural nodule, necessitating the exclusion of uterine or gastrointestinal metastases via immunohistochemical and molecular profiling. The glands exclusively express cytokeratin epithelial markers, whereas the stromal cells demonstrate strong positive vimentin expression. To date, only three cases of ovarian mucinous tumors associated with carcinosarcomatous mural nodules have been reported, with the histological types of the ovarian mucinous tumors identified as mucinous cystadenoma, mucinous carcinoma, and borderline mucinous tumor (15–19) (Table 1). For carcinosarcoma management, chemotherapy is the predominant treatment approach; however, due to insufficient follow-up periods (10 and 24 months), the prognosis remains undetermined.

**Table 1:** Summary of the cases of ovarian mucinous tumor with carcinosarcomatous mural nodule.

Characteristic	Present case (2026)	Chang et al. (17)	Søndergaard et al. (19)	Bruijn et al. (18)
Case	1	1	1 (Case 3)	1
Epithelial ovarian tumor	Borderline mucinous	Borderline mucinous	Mucinous cystadenoma	Mucinous carcinoma
Mural nodule	Carcinosarcoma	Carcinosarcoma	Carcinosarcoma	Carcinosarcoma
Age	27	29	29	27
FIGO stage	Ia	Ia	I	Ia
Therapy	TC	USO + ChT	RH	USO + Omen
Follow-up	NED,5.5 months	NED, 10 months	NED, 24 months	/
Nodule size	1–3.5 cm (multifocal)	5 × 4 × 2 cm in greatest dimension (multifocal)	/	/
Diagnostic method	Dual staining/Immunohistochemistry	Immunohistochemistry	/	Immunohistochemistry

This article presents a case of ovarian borderline mucinous tumor with carcinosarcomatous mural nodules in a 27-year-old female patient. The patient underwent exploratory laparotomy combined with fertility-sparing ovarian cancer staging surgery. Although the patient was staged as FIGO IA without evidence of extra-ovarian spread, the presence of carcinosarcomatous mural nodules warranted adjuvant chemotherapy. Unlike sarcoma-like nodules, malignant mural nodules (including carcinosarcomatous and anaplastic types) are associated with aggressive clinical behavior and poor prognosis (20). Consequently, management strategies often align with those for invasive ovarian carcinoma rather than borderline tumors (21). Given the high-risk histological features, the patient received six cycles of paclitaxel and carboplatin (TC regimen), consistent with reported case series managing malignant mural nodules to mitigate recurrence risk (22). During a 5.5 months follow-up, no disease recurrence was observed. The tumor exhibited multifocal mural nodules with grayish-yellow cut surfaces, solid consistency, and clear demarcation from surrounding tissues. The carcinomatous component consisted of moderately to poorly differentiated glands, while the sarcomatous component was composed of abundant short spindle-shaped cells.

Notably, although vascular invasion was not evident in the sarcomatous component, its malignant nature was indicated by high expression of p53 and Ki67, along with scattered CD68-positive histiocytes (23). IHC results demonstrated distinct lineages but presented challenges in specific subtyping. Epithelial component: The glandular epithelial areas within the nodule expressed CK(Pan), CK7, EMA, and villin. Mesenchymal (spindle cell) component: The spindle cell areas were positive for vimentin, SMA (partial). However, markers for specific sarcomatous differentiation were negative or non-diagnostic, including desmin, myogenin, CD34, SATB2, S100, HMB45, calponin, ER, PR, WT1 and PAX-8. While the IHC profile confirms the biphasic nature (carcinosarcomatous) of the nodule, the lack of specific differentiation markers (negative desmin and myogenin arguing against rhabdomyosarcoma; negative CD34 arguing against solitary fibrous tumor) suggests an undifferentiated sarcoma phenotype rather than a specific histological subtype. Consequently, while the presence of malignant epithelial and mesenchymal components was confirmed, immunohistochemical analysis revealed that the sarcomatous component expressed only non-specific mesenchymal markers without evidence of specific lineages. This aligns with documented cases of malignant mural nodules arising in borderline ovarian tumors, where sarcomatous elements frequently display undifferentiated characteristics (24). Therefore, we have diagnosed this case as a borderline mucinous tumor of the ovary with carcinosarcomatous mural nodules, explicitly acknowledging the non-specific nature of the sarcomatous component. It is worth noting that the classification of mucinous tumors with malignant mural nodules remains a subject of discussion. While Blaustein’s Pathology of the Female Genital Tract suggests that such tumors are best classified as variants of mucinous carcinoma or carcinosarcoma due to their aggressive behavior, the WHO Classification of Tumours (24) recommends retaining the background diagnosis when the predominant component is borderline. In the present case, given that the majority of the tumor exhibited borderline features with carcinosarcomatous nodule, we adopted the diagnosis to accurately reflect the histological heterogeneity and potential histogenesis.

Additionally, CK(Pan) immunohistochemical staining revealed rare scattered positive cells within the spindle cell stroma. The histogenesis of carcinosarcomas remains contentious, primarily centered on the collision, conversion, and combination hypotheses (25). Among these, the collision and conversion models are the most widely recognized. The collision theory posits that carcinomatous and sarcomatous elements arise from independent clonal origins and subsequently merge. Conversely, the conversion theory suggests sarcomatous differentiation arising from a pre-existing carcinoma. Alternatively, the combination theory proposes a common monoclonal progenitor for both distinct components. To further explore this issue, we performed CK(Pan)/vimentin immunohistochemical double staining, which demonstrated that the carcinomatous component expressed CK(Pan) exclusively while the sarcomatous component expressed vimentin, with focal CK(Pan) positivity also observed within the spindle cell component. Due to the cytoplasmic localization of both markers, visualizing a distinct co-expression pattern was challenging. Nevertheless, CK(Pan) positivity was definitively identified in the spindle cell area. The co-expression of CK(Pan) and vimentin in the spindle cell area is a critical diagnostic feature. While the spindle cells were predominantly vimentin-positive, the presence of scattered CK-positive cells revealed by immunohistochemical single and dual staining supports the hypothesis of epithelial-mesenchymal transition (EMT) rather than a collision tumor (26). This finding aligns with molecular studies suggesting a monoclonal origin, where the malignant nodule arises from the borderline epithelium through dedifferentiation, often driven by TP53 mutations in addition to the background KRAS/BRAF alterations (27). Recognizing these features is vital for differential diagnosis, particularly to distinguish from pure ovarian sarcomas or metastatic carcinomas, which carry different prognostic implications. Patients with malignant mural nodules generally have a prognosis intermediate between borderline tumors and high-grade carcinomas, necessitating careful long-term follow-up. To our knowledge, this immunohistochemical double-staining approach for characterizing mural nodules in ovarian mucinous tumors has not been previously reported in the literature. Unlike conventional single-stain immunohistochemistry, which requires serial sections and may suffer from tissue heterogeneity, dual staining allows for the simultaneous visualization of epithelial and mesenchymal markers on the same tissue section and at the single-cell level. This is critical for confirming the true biphasic nature of a carcinosarcomatous nodule. A key diagnostic challenge is distinguishing true carcinosarcoma from sarcomatoid carcinoma or spindle cell carcinoma (28). The dual staining method clearly delineates whether CK and vimentin expression occurs in distinct cell populations (supporting carcinosarcoma) or shows co-expression within the same cells (supporting sarcomatoid carcinoma), thereby reducing diagnostic ambiguity.

## 4 Conclusion

Ovarian borderline mucinous tumors with carcinosarcomatous mural nodules represent a rare entity with aggressive potential that challenges standard borderline tumor management. This case highlights the diagnostic utility of CK(Pan)/vimentin immunohistochemical double staining in confirming the biphasic nature of mural nodules and supporting a monoclonal origin via epithelial-mesenchymal transition, thereby reducing ambiguity in distinguishing true carcinosarcoma from sarcomatoid carcinoma. Despite an early FIGO IA stage, the presence of malignant nodules necessitated adjuvant chemotherapy following fertility-sparing surgery, reflecting a treatment strategy aligned with invasive carcinoma rather than borderline neoplasms. While short-term follow-up revealed no recurrence, the historically poor prognosis associated with malignant mural nodules mandates rigorous long-term surveillance. Ultimately, integrating dual-marker staining into routine pathological assessment and establishing standardized adjuvant therapy protocols through further multicenter research are essential to optimize clinical outcomes for this high-risk patient population.

## Data Availability

The original contributions presented in the study are included in the article/supplementary material, further inquiries can be directed to the corresponding author.
